# Supplementary material for: Predator-Prey Interactions between Halobacteriovorax and Pathogenic Vibrio parahaemolyticus Strains: Geographical Considerations and Influence of Vibrio Hemolysins
Source: Microbiol Spectr. 2023 Jul 6;11(4):e02353-23. doi: 10.1128/spectrum.02353-23 (PMC10434201; doi:10.1128/spectrum.02353-23)
Supplement: Supplemental file 1 — Table S1. Download spectrum.02353-23-s0001.docx, DOCX file, 0.01 MB [file spectrum.02353-23-s0001.docx]

**Supplemental Table S1. Partial sequences of 16S rRNA genes for four *Halobacteriovorax* strains used in this study**

**Strain S11**

CTCTGTCAGATAGGAAGAATTGCCAGGGGTCCAATAGGCCCTTGGAGTGACGGTACTATCAAAGGAAGCACCGGCTAACTTCGTGCCAGCAGCCGCGGTAATACGAAGGGTGCAAGCGTTGTTCGGATTTATTGGGCGTAAAGCGCGCGCAGGCGGGCAATTAAGTCAGATGTGAAATCTCGGGGCTCAACCCCGAAACTGCGTCTGAAACTGGTTGCCTAGAGTTTCAGAGGGGAGAGCGGAATTTCGCATGTAGGGGTAAAATCCGTAGAGATGCGAAGGAACACCAGAGCCGAAGGGGGCTCTCTGGCTGACAACTGACGCT

**Strain G3**

CTCTGTCAGATGGGAAGAACAGCAGTTGGTCCAATAGGCCATCTGTTTGACGGTACCTTCAAAGGAAGCACCGGCTAACTTCGTGCCAGCAGCCGCGGTAATACGAAGGGTGCAAGCGTTGTTCGGATTTACTGGGCGTAAAGCGCGCGCAGGCGGATTGATAAGTCAGATGTGAAATCTCGGGGCTCAACCCCGAAACTGCGTCTGAAACTGTTGATCTAGAGTTATGTAGGGGAGAAGGGAATTTCGCATGTAGGGGTAAAATCCGTAGAGATGCGAAGGAACACCAGAGCCGAAGGGGCTTCTCTGGGCATAAACTGACGCT

**Strain H4**

CTCTGTCAGATAGGAAGAATTGCCAAGGGTCCAATAGGCCCTTGGAGTGACGGTACTATCAAAGGAAGCACCGGCTAACTTCGTGCCAGCAGCCGCGGTAATACGAAGGGTGCAAGCGTTGTTCGGATTTATTGGGCGTAAAGCGCGCGCAGGCGGGCAGCTAAGTCAGATGTGAAATCTCGGGGCTCAACCCCGAAACTGCGTCTGAAACTGGTTGCCTAGAGTTTCAGAGGGGAGAGCGGAATTTCGCATGTAGGGGTAAAATCCGTAGAGATGCGAAGGAACACCAGAGCCGAAGGGGGCTCTCTGGCTGACAACTGACGCT

**Strain OS1**

CTCTGTCAGATAGGAAGAATTGCCAAGGGTCCAATAGGCCCTTGGAGTGACGGTACTATCAAAGGAAGCACCGGCTAACTTCGTGCCAGCAGCCGCGGTAATACGAAGGGTGCAAGCGTTGTTCGGATTTATTGGGCGTAAAGCGCGCGCAGGCGGGCAATTAAGTCAGATGTGAAATCTCGGGGCTCAACCCCGAAACTGCGTCTGAAACTGGTTGCCTAGAGTTTCAGAGGGGAGAGCGGAATTTCGCATGTAGGGGTAAAATCCGTAGAGATGCGAAGGAACACCAGAGCCGAAGGGGGCTCTCTGGCTGACAACTGACGCT
